# Health Risk Assessment for Exposure to Nitrate in Drinking Water in Central Java, Indonesia

**DOI:** 10.3390/ijerph18052368

**Published:** 2021-03-01

**Authors:** Callum Lowe, Johanna Kurscheid, Aparna Lal, Ross Sadler, Matthew Kelly, Donald Stewart, Budi Laksono, Salvador Amaral, Darren Gray

**Affiliations:** 1 Department of Global Health, Research School of Population Health, Australian National University, Acton 2601, Australia; johanna.kurscheid@anu.edu.au (J.K.); matthew.kelly@anu.edu.au (M.K.); donald.stewart@griffith.edu.au (D.S.); salvadoramaralcoro@gmail.com (S.A.); darren.gray@anu.edu.au (D.G.); 2 Swiss Centre for International Health, Swiss Tropical and Public Health Institute, 4051 Basel, Switzerland; 3 National Centre for Epidemiology and Population Health, Research School of Population Health, Australian National University, Acton 2601, Australia; aparna.lal@anu.edu.au; 4 School of Public Health, Griffith Health, Griffith University, South Brisbane 4111, Australia; ross.sadler@griffith.edu.au; 5 School of Medicine, Griffith Health, Griffith University, South Brisbane 4111, Australia; 6 Yayasan Wahana Bakti Sejahtera (YWBS) Foundation, Semarang 50183, Indonesia; dokterbudilaksono@gmail.com

**Keywords:** health risk assessment, nitrate, birth defects, drinking water, Indonesia

## Abstract

Since 2005, over 30 epidemiological studies have evaluated the association between nitrate in drinking water and adverse health outcomes. Conditions that lead to nitrate pollution in water, such as open defecation, the proximity of septic tanks to water sources, and the use of inorganic fertilizer, are rampant in Indonesia, which has experienced little research evaluating nitrate in drinking water. We conducted a health risk assessment for exposure to nitrate in drinking water and evaluated the nitrate concentration in key water sources in two villages of rural Central Java, Indonesia. The nitrate concentrations in the drinking water ranged from 3.55 mg/L to 26.75 mg/L as NO_3_^−^. Daily nitrate intake estimates, calculated at 50% and 95% exposure to the maximum nitrate concentration of the drinking water in both villages, were above the levels associated with birth defects, colorectal cancer, and thyroid conditions observed in other studies. There was a large variation in nitrate concentrations between and within the villages at different water sources. Further research into whether these health outcomes exist in rural Central Java, Indonesia will be required to better understand this risk.

## 1. Introduction

There is growing concern over the health risks of elevated levels of nitrate (NO_3_^-^) in drinking water—more than 30 epidemiological studies have evaluated the association between nitrate in drinking water and adverse health outcomes since 2005 [1]. Nitrate consumption leads to the formation of nitrite in the stomach, which is a precursor for N-nitroso compounds (NOCs) that are known to be teratogens and carcinogens [2]. The ingestion of water with elevated nitrate concentrations is consistently associated with colorectal cancer, birth defects, and thyroid cancer.

In Spain and Italy, Espejo-Herrera et al. (2016) found that participants in a case-control study with a daily nitrate intake from water greater than 10 mg had higher odds (OR (odds ratio) 1.49, 95% CI (confidence interval) = 1.24–1.78) of colorectal cancer compared to those consuming < 5 mg nitrate per day [3]. Birth defects are also reported to correlate with maternal nitrate intake. Brender et al. (2013) conducted a case-control study which found that mothers of offspring with limb deficiencies, cleft palate, and cleft lip were, respectively, 1.8 (95% CI = 1.3–3.1, *p* < 0.05), 1.9 (95% CI = 1.2–3.1, *p* < 0.05) and 1.8 (95% CI = 1.1–3.1, *p* < 0.01) times more likely than the control mothers to ingest greater than 5.42 mg of nitrate daily (vs. <1 mg) [4]. A similar study in Canada found an increased risk of congenital anomalies among mothers ingesting water with nitrate concentrations between 4.42 and 24.58 mg/L (vs. <4.42 mg/L, OR = 2.25, 95% CI = 0.92–5.52) [5]. In the United States, Ward et al. (2010) observed that in a cohort of older women, those ingesting well water with nitrate concentrations above 22.1 mg/L for 5+ years had a higher risk of thyroid cancer (RR = 2.6, 95% CI = 1.1–6.2) compared to women with no exposure to well water [6]. A study of subclinical hypothyroidism among a sample of an Amish community in Pennsylvania found an increased risk among women who ingested water with nitrate concentrations above 28.73 mg/L (vs. < 28.73 mg/L, OR = 1.6, 95% CI = 1.11–2.32) [7].

Surface water, such as that from springs and rivers, is subject to environmental contamination, and is the water source for 18% of the people in Indonesia [8]. Approximately 10% of the Indonesian population practice open defecation [9], but among rural communities this rate is as high as 55% [10]. Given the presence of nitrogen compounds in urine and feces, open defecation contributes to nitrate in the drinking water [11]. Where septic tanks are located close to water wells in Indonesia, a greater risk of nitrate contamination in well water is observed [12]. Application of inorganic fertilizer for agricultural activities in Indonesia has been associated with elevated levels of nitrate in the groundwater [13]. The nitrate concentrations in well water in Indonesia have been reported as high as 82 mg/L, implying a low risk for methemoglobinemia and an elevated risk for birth defects [14]. A case-control study of Indonesian hospital patients by Fathmawati et al. (2017) found that patients consuming nitrate-contaminated well water above 50 mg/L were at nearly three times higher risk of colorectal cancer (OR = 2.82, 95% CI = 1.075–7.395, *p* < 0.05, adjusted for confounders including protein intake) compared to those who consumed water below 50 mg/L, and this risk was higher for those with >10 years exposure (OR = 4.31, 95% CI = 1.32–14.1, *p* < 0.05) [15].

Overall, knowledge of nitrate concentration in drinking water and its associated health risks in Indonesia is lacking. This study is, to the authors’ knowledge, the first to report nitrate concentrations in rural Indonesian household tap water as well as the water supply network in rural Indonesian villages. The main aim of the current study was to quantify nitrate levels across two villages in Central Java, and to conduct a preliminary health risk assessment of nitrate in the drinking water for these populations.

## 2. Methods

### 2.1. Study Area

The present study was conducted in two villages in Wonosobo, Central Java, Indonesia, namely Desa (village) Losari and Desa Topengan. These villages are located on the foothills of Mount Sindoro and Mount Bismo, respectively, and are currently acting as control villages in a large-scale latrine (“BALatrine”) intervention trial [10]. Average precipitation in Wonosobo is higher in February (wet season) compared to August (443 mm vs. 93 mm rainfall) [16]. The area surrounding these villages is primarily used for horticultural purposes. Topengan is situated on a steeper incline than Losari. In Topengan, there is one spring piped approximately two kilometers up the face of Mount Bismo to one holding tank where many smaller pipes provide a water source to households in Topengan. Multiple springs with underground and above-ground pipes provide water to houses in Losari. In both villages, household water drains away in concrete channels, sometimes via fishponds, and then into the village fields below for farm use and then finally to a river. Both villages are near rivers. No households have an improved latrine installed.

### 2.2. Sampling Procedure

In October 2018, preliminary data aimed at identifying key water sources entering/exiting households were obtained. These included springs, holding tanks, household taps, fishponds, and concrete outflow channels. Five households from each village were randomly selected for the sampling of household tap water. For household taps, one tap that was located in or near the food preparation area of each house was sampled. To account for seasonal variation, water sampling was performed in February (wet season) and August (dry season) of 2019. Only results pertaining to the wet season are used in this analysis as the dry season results are to be used in future analysis. Crude data from the wet season and the dry season are presented in Table S1, supplementary material section.

The water samples were collected at midday. The taps were run for 2–3 min or until the water temperature was stable, after which time a sample was collected and stored as per the procedure outlined in Section 2.2 of Sadler et al., 2016. The water samples were stored and transported frozen up until flow injection analysis (FIA). The equipment used was a Lachat QC8500 and the colorimetric method was a cadmium reduction.

### 2.3. Quality Control

Water samples for the nitrate analysis were stored at −20 °C until they were transported to Australia and analyzed by the Queensland Health Forensic and Scientific Services, which holds National Association of Testing Authorities (NATA) accreditation for nitrate analysis in water.

### 2.4. Statistical Analysis

Nitrate concentrations were first converted from mg/L as nitrogen to mg/L as nitrate by multiplying the concentration in mg/L as nitrogen by the molar mass of nitrate divided by the molar mass of nitrogen. From here on, all nitrate concentrations pertaining to data collected in this study are reported as mg/L as nitrate (NO_3_^−^).

The mean nitrate concentrations of samples from the same water source for each village were calculated. Following this, the five household tap water samples from each village were used in the remaining analysis. First, the cumulative probability distribution of the household tap nitrate concentrations was calculated using the formula:

(1) $${\mathrm{CP}}_{\mathrm{EXP}}\left(\%\right)=\;\frac{i}{n}\times 100$$

whereby CP_EXP_ is the cumulative probability at a given exposure; i, the ith sample (arranged by ascending nitrate concentration); and n, the total number of samples in each village (n = 5). This formula was adapted from Sadler et al. (2016) [14]; however, we chose to set n as the denominator, as opposed to n + 1, due to the small sample size used. Using the cumulative probabilities, a cumulative probability distribution (CPD) plot was created for each village. A linear regression line was fitted, allowing for the calculation of two important values for each village—CP_EXP50_ and CP_EXP95_. These two values represent the nitrate concentration of household taps, which reached a cumulative probability of 50% and 95%, respectively. Calculation of these two exposure values means that the risk for nitrate in household tap water is assessed at median levels (CP_EXP50_) and at a conservative level (CP_EXP95_).

The mean and standard deviation of the nitrate concentration of household taps within each village was also calculated. Then, a two-sided t-test for equal means was performed, assuming equal variance after determining from an F-test if equal variance was not significant.

Because nitrate intake is a function of both the concentration of nitrate in the water and the amount of water consumed, which is dependent on physiology, it is necessary to obtain estimates of this parameter. The H4H (Hydration for Health) Hydration Calculator was used to obtain daily water intake requirements, given height and weight [17]. These estimates are based on the recommended intakes. Specific details of this calculation including weight and height inputs based on the Indonesian population have been reported elsewhere [14]. For pregnant women, water intakes and expected weight are calculated at the first trimester because the birth defect effects of nitrate have been reported to be induced in the first trimester of pregnancy [4].

To calculate an estimate of the daily intake dose of nitrate from water based on the recommended water intakes, nitrate concentrations from household tap water (mg/L) were converted into daily intakes using the below equation [14]:

(2) $$\mathrm{DID}\left(\mathrm{mg}/\mathrm{kg}.\mathrm{bw}/\mathrm{day}\right)=\;\frac{C\times \mathrm{IR}}{\mathrm{BW}}$$

where DID is the daily intake dose; C, concentration (mg/L) of nitrate; IR, ingestion rate of water (L/day), as previously described; and BW, average body weight (kg). The estimated daily intake dose of nitrate from water was calculated at two exposures (nitrate concentrations), namely CP_EXP50_ and CP_EXP95_.

### 2.5. Ethics

The Australian National University Human Research Ethics committee supplied ethical approval for the study (protocol 2017/038).

## 3. Results

The demographic and geographic characteristics of the villages in the study and of the overarching territory containing the villages are presented in Table 1. Losari and Topengan had 270 and 193 houses, respectively, and the majority of adults in Losari were farmers (63.0%). The majority of the land is used for farming in both villages; however, only data pertaining to the overarching territory of Topengan (Desa Sitiharjo) was available.

**Figure 1:**
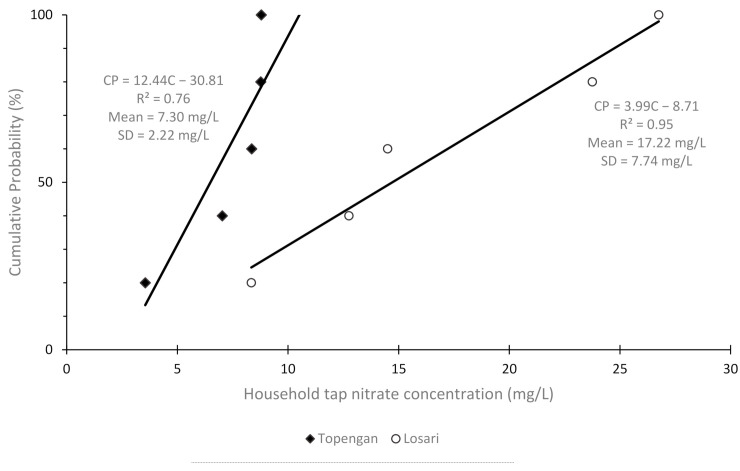
Cumulative probability (CP) distribution plot of household tap nitrate concentrations (C) in Losari and Topengan. SD = Standard Deviation.

The CP_EXP50_ and CP_EXP95_ values were 14.71 and 25.99 mg/L, respectively, in Losari and 6.5 and 10.11 mg/L, respectively, in Topengan. Mean nitrate concentration among household taps in Topengan and Losari was 7.30 ± 2.22 mg/L and 17.22 ± 7.74 mg/L, respectively (Figure 1). A two-sided t-test for equal means given equal variance (*p*-value from F-test of equal variance = 0.07) returned *p* = 0.02, indicating a significant difference in the mean nitrate concentrations of household taps between villages.

**Figure 2:**
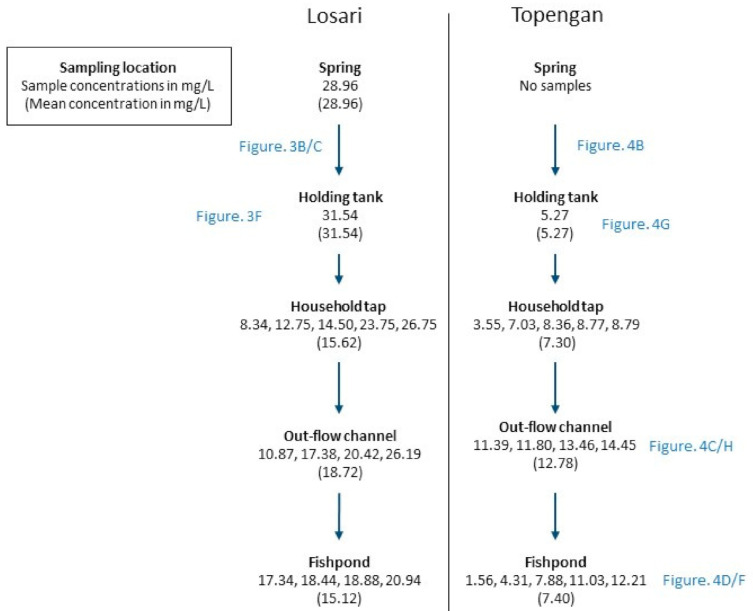
Flowchart of nitrate concentrations in the key water sources in Losari and Topengan. See Figure 3 and Figure 4 for colored photographs of sampling locations.

**Figure 3:**
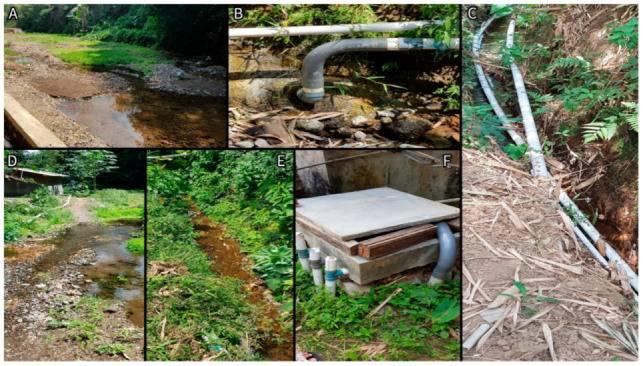
Flow of water in Losari. (**A**,**D**,**E**)—Neighboring environment of groundwater spring. (**B**,**C**)—Pipes from spring to holding tank. (**F**)—Water holding tank.

**Figure 4:**
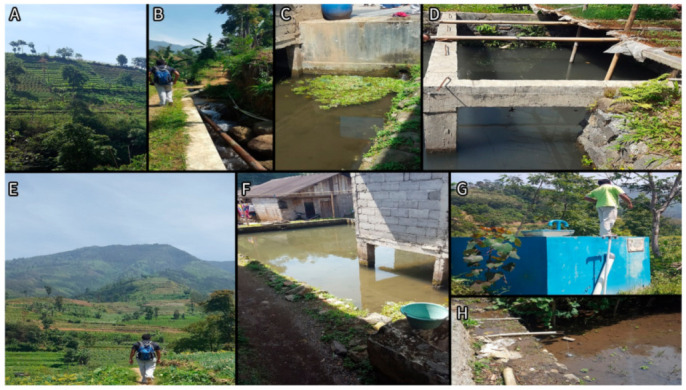
Flow of water in Topengan. (**A**,**E**)—Surrounding environment of Topengan village. (**B**)—Pipes from the spring to the holding tank. (**C**)—Concrete outflow channel to the fishpond. (**D**)—Crops grown above the fishpond. (**F**,**H**)—Fishpond. (**G**)—Water holding tank.

Nitrate concentrations were consistently higher in Losari compared to Topengan (Figure 2). In Losari, the nitrate concentrations of the spring and the holding tank samples were slightly different (28.96 mg/L and 31.54 mg/L, respectively), and were, on average, much lower in household taps (mean of 15.62 mg/L). In Topengan, household tap water generally had higher nitrate concentrations than the holding tank. In both villages, there was little difference in the mean nitrate concentrations between the household tap water samples and the fishpond water samples. Nitrate concentrations were slightly higher in the water from outflow channels compared to the fishponds in both villages. Photographs of the water sampling locations are present in Figure 3 and Figure 4.

The estimated nitrate intake from drinking water was as high as 96.16 mg/day for adult males and pregnant females when calculated at CP_EXP95_ in Losari, and as low as 5.07 mg/day for infants when calculated at CP_EXP50_ in Topengan (Table 2). When the estimated nitrate intakes were divided by the estimated average weight for each demographic group, infants had higher weight-adjusted intakes than adults, reaching 6.03 mg/kg/day when calculated at CP_EXP50_ in Losari. The lowest weight-adjusted estimate was for adult males at 0.41 mg/kg/day when calculated at CP_EXP50_ in Topengan.

## 4. Discussion

This study found that estimates of daily nitrate intake from drinking water varied between households in Wonosobo, Indonesia. Whilst levels did not exceed the 50 mg/L guideline value associated with infant methemoglobinemia, they were as high as levels reported elsewhere in the last decade as having an association with adverse health outcomes, including birth defects, colorectal cancer and thyroid conditions. A separate study in the same villages carried out by the lead author indicated that the water from household taps is frequently boiled and consumed as tea, coffee, or plain water (author observation). Although no estimates of water intake can be derived and used to compare to the estimates used in this study, it is assumed that there is some amount of risk associated with drinking this water.

We noticed several potential sources of nitrate contamination at the study sites. The overwhelming majority of the households in both villages did not have an improved latrine installed (Table 1), and as such, open defecation is likely to be prevalent, which contributes to nitrate pollution [11]. These villages have intensive horticultural activities, as the majority of land is used for farming (Table 1), which could potentially cause nitrate contamination from fertilizer use [13]. Rubbish and sacks of fertilizer were observed surrounding the Losari spring environment (author observation). Some homeowners grew crops above water sources (Figure 4D), which would contribute to nitrate contamination where fertilizer is used.

It has been shown that pregnant mothers in the United States have a higher risk of giving birth to a child with birth defects when daily nitrate intakes exceed 5.42 mg [4]. This value is easily exceeded given that the levels of nitrate in a single liter of tap water exceed 5.42 mg at CP_EXP50_ in both villages. Nitrate concentrations in water above 4.42 mg/L consumed by pregnant women were associated with an increased risk of giving birth to a child with congenital anomalies in a Canadian study [5]. This would be exceeded by all the samples in Losari and by many in Topengan (Figure 2). In Indonesia, congenital malformations (a type of birth defect) made up 1.4% of deaths for infants in the first six days of life, and 19% of deaths for infants in the age group aged 7–28 days [20]. A direct investigation into the associations between elevated nitrate intake and congenital malformations in rural Indonesian communities would be useful for better understanding the potential role of elevated nitrate intake.

Levels of nitrate concentration in drinking water, as well as daily nitrate intakes that have an association with adverse health outcomes in the literature, were exceeded in this study. For example, daily intakes above 10 mg have been associated with an increased risk of colorectal cancer [3], which would be exceeded by the adults in our study at CP_EXP50_ (Table 2). This association of elevated nitrate intake and colorectal cancer has also been demonstrated in Indonesia [15]. Colorectal cancer is one of the ten most common cancers in Indonesia [21]. Further research is needed to investigate whether areas with high rates of colorectal cancer also report elevated nitrate concentrations in the drinking water.

Elevated nitrate concentrations in water have been associated with adverse conditions of the thyroid. One North American study by Ward et al. (2010) found women exposed to water with nitrate concentrations above 22.1 mg/L for 5+ years to be at a higher risk of thyroid cancer [6]. This concentration would be exceeded by two household tap water samples in Losari (Figure 2), indicating a potential health risk. Hypothyroidism was associated with the consumption of water with nitrate concentrations above 28.73 mg/L in a study by Aschebrook-Kilfoy et al. (2012) [7], which is only 2 mg/L higher than the highest nitrate concentration in Losari (Figure 2).

We observed a significantly higher mean nitrate concentration in the household tap water samples in Losari compared to Topengan. One of the striking differences between the two villages was that the location of the spring in Topengan was at a high altitude up a mountain slope, whereby groundwater near the spring was subject to less environmental nitrate contamination than in Losari. Furthermore, the groundwater spring in Losari (see Figure 3A/D) was unsealed and appeared to have the potential for contamination with surface water from the surrounding environment, in which litter was spotted (author observation). Smith et al. (2000) noted that villages on Lombok island of Indonesia that were situated on the slopes of Mount Rinjani had fast-flowing groundwater due to the slope of the land, and consequently had lower nitrate concentrations in the groundwater [22]. A similar phenomenon may explain the differences in nitrate concentrations between the two villages in our study. The mean nitrate concentration of household taps in Losari was half the nitrate concentration reported in the holding tank in Losari yet was slightly higher for Topengan (Figure 2). Further research with more frequent sampling across a larger variety of villages is required to better understand this difference.

Many epidemiological studies evaluate the exposure to nitrate in water through historical readings of water near an individual’s place of residence, whilst our study is based on primary on-site water sampling. In calculations of daily nitrate ingestion from drinking water, the contributions of other water sources (e.g., bottled water, soft drinks, flavored drinks, etc.) towards the daily water intake requirement were not considered. As such, there is the potential that the daily nitrate ingestion values are overestimated if other beverages contributing to daily water intake have lower nitrate concentrations than that of the tap water in this study. It should also be considered, however, that we did not measure dietary nitrate ingestion. Therefore, when the literature values report high levels of nitrate ingestion from both oral and dietary sources as having an association with adverse health outcomes, it is still plausible that this would be exceeded with the tap water samples in this study. There was large within-sampling site variation in nitrate concentrations from most water source types. This may be due to the inconsistencies in the water distribution networks between households, but further research is required to account for this variation. However, our study is strengthened by a diverse range of water sampling locations, which add to our assessment and demonstrate the plausibility of the household tap nitrate concentrations observed. Furthermore, we directly collected and analyzed water samples for nitrate as opposed to utilizing historical institutional water analysis data; thus, we can confirm that the people in the villages were indeed exposed to tap water with the nitrate concentrations we reported. This is, to the authors’ knowledge, the first health risk assessment for nitrate in rural Central Java, Indonesia that reports nitrate concentrations at various water sources in a rural village.

Bottled water is the most reliable source of water in Indonesia, but it is only consumed by those who can afford it [23]. The commercialization of bottled water has meant that the use of groundwater, such as that used in the villages of this study, is insufficiently regulated [23]. Wastewater is neither collected nor treated in rural Indonesia [24], and the results of government water testing are not made public. The distribution and collection of water in the villages of this study were managed by the local villages (author observation). These factors create an environment whereby pollutants in the water such as nitrate are not quantified or controlled, and our results indicate the urgent need for further investigation into the sources and risks associated with nitrate in tap water.

## 5. Conclusions

Estimated nitrate intakes from household tap water based on recommended water intakes from two villages in Central Java, Indonesia, were at and above levels associated with adverse health conditions, particularly towards birth defects, colorectal cancer, and the thyroid gland. Nitrate concentrations from key water sources were overall higher in Losari compared to Topengan, which may be due to the positioning of the spring in Topengan on an elevated mountain slope. Further research is required to better understand the changes in nitrate concentration observed in the study between water sources. Whether these elevated nitrate levels observed have an association with adverse health conditions in this region of the world would make for interesting further research.

## Figures and Tables

**Table 1:** Demographic and geographic characteristics of the study sites.

**Characteristic**	**Village**	
	*Losari*	*Topengan*
Number of households	270	193
Number of households with improved latrine installed	7	1
Distance to district centre	14.6 km	9 km
Distance between villages	23.4 km	
Proportion of adults that are farmers	63.0%	43.3%
	Territory/Region containing village *^,†^	
	*Desa Candimulyo*	*Desa Sitiharjo*
Total area (hectares)	413 ha	351 ha
Area of land used for farming	n/a	329 ha
Total population	5779	3730
Infant males (0–4 years)	270	157
Infant females (0–4 years)	285	164
Adult males (20+ years old)	1809	1204
Adult females (20+ years old)	1904	1016

* Villages in rural Central Java are referred to as “Dusun”, whilst the overarching village containing several “Dusun” is referred to as “Desa”, which is the smallest geographical division in which routine statistics are collected. ^†^ Statistical data obtained from government routine statistics publication [18,19].

**Table 2:** Estimated nitrate intakes among infants and adults calculated at CP_EXP50_ and CP_EXP95_ for each village.

Sub-Population	Recommended Water Intake (L) *	Estimated Weight ^†^	Nitrate Intake Unit	Nitrate Intake			
				Losari		Topengan	
				CP_EXP50_	CP_EXP95_	CP_EXP50_	CP_EXP95_
Infants (birth)	0.78	3.36	mg/day	11.47	20.27	5.07	7.89
			mg/kg. body weight/day	3.41	6.03	1.51	2.35
Infants (3 months)	1.181	5.76	mg/day	17.37	30.69	7.68	11.94
			mg/kg. body weight/day	3.02	5.33	1.33	2.07
Adult males	3.7	59	mg/day	54.43	96.16	24.05	37.41
			mg/kg. body weight/day	0.92	1.63	0.41	0.63
Adult non-pregnant females	3.4	53	mg/day	50.01	88.37	22.10	34.37
			mg/kg. body weight/day	0.94	1.67	0.42	0.65
Pregnant females (end of 1st trimester)	3.7	48.5	mg/day	54.43	96.16	24.05	37.41
			mg/kg. body weight/day	1.12	1.98	0.50	0.77

* Recommended water intake calculated using the estimated water intake requirements for each sub-population from H4H Hydration Calculator (H4H Hydration Calculator, 2016). ^†^ Estimated weights obtained from Sadler et al., 2016.

## Data Availability

The data presented in this study are available in supplementary material Table S1.

## Supplementary Materials

The following are available online at https://www.mdpi.com/1660-4601/18/5/2368/s1, Table S1: Crude nitrate concentrations from wet and dry season sampling.
