# Supplementary material for: Health Risk Assessment for Exposure to Nitrate in Drinking Water in Central Java, Indonesia
Source: Int J Environ Res Public Health. 2021 Mar 1;18(5):2368. doi: 10.3390/ijerph18052368 (PMC7957596; doi:10.3390/ijerph18052368)
Supplement: Supplementary file 1 [file ijerph-18-02368-s001.pdf]

Supplementary material

Table S1. Crude nitrate concentrations from wet and dry season sampling.

**Losari**	**Nitrate concentration (mg/L)**	
	Wet season	Dry season
Spring 1	28.9608	27.28
Holding Tank 1	31.5392	27.72
Fish pond 1	18.436	27.28
Out flow channel 1	17.38	22
Fish pond 2	20.944	22.88
House Hold tap 1	14.5024	14.08
House hold tap 2	26.752	25.52
Out flow channel 2	20.4204	22.44
Fish pond 3	18.876	23.32
House Hold tap 3	23.7512	23.32
House Hold tap 4	8.3424	12.32
Fish pond 4	17.336	18.04
Out flow channel 3	26.1932	13.64
House Hold tap 5	12.7512	17.16
Out flow channel 4	10.868	3.564
**Topengan**		
Holding Tank 1	5.2712	10.12
House Hold tap 1	7.0268	10.56
House Hold tap 2	3.5464	8.8
House Hold tap 3	8.3556	10.12
House Hold tap 4	8.7692	10.12
House Hold tap 5	8.7912	10.12
Fish pond 1	11.0308	7.92
Fish pond 2	4.312	0.0968
Fish pond 3	1.5576	7.48
Fish pond 4	7.8848	8.8
Fish pond 5	12.21	8.36
Out flow channel 1	11.8008	7.04
Out flow channel 2	11.3872	9.68
Out flow channel 3	14.4496	8.36
Out flow channel 4	13.4596	5.72
